# Documenting the microbiome diversity and distribution in selected fleas from South Africa with an emphasis on the cat flea, *Ctenocephalides f. felis*.

**DOI:** 10.1017/S0031182023000835

**Published:** 2023-09

**Authors:** Conrad A. Matthee, Anandi Bierman, Boris R. Krasnov, Sonja Matthee, Luther van der Mescht

**Affiliations:** 1 Department of Botany and Zoology, Evolutionary Genomics Group, Stellenbosch University, Stellenbosch, South Africa; 2 Department of Conservation Ecology & Entomology, Centre for Invasion Biology, Stellenbosch University, Stellenbosch, South Africa; 3 Mitrani Department of Desert Ecology, Swiss Institute for Dryland Environmental and Energy Research, Jacob Blaustein Institutes for Desert Research, Ben-Gurion University of the Negev, Midreshet Ben-Gurion, Israel; 4 Department of Conservation Ecology & Entomology, Stellenbosch University, Stellenbosch, South Africa

**Keywords:** *Bartonella*, *Ctenocephalides canis*, *Ctenocephalides connatus*, *Ctenocephalides felis*, microbiome, *Rickettsia*, South Africa, 16S rRNA metagenetic

## Abstract

The factors that influence parasite associated bacterial microbial diversity and the geographic distributions of bacteria are not fully understood. In an effort to gain a deeper understanding of the relationship between the bacterial diversity of *Ctenocephalides* fleas and host species and the external environment, we conducted a metagenetic analysis of 107 flea samples collected from 8 distinct sampling sites in South Africa. Pooled DNA samples mostly comprising of 2 or 3 individuals sampled from the same host, and belonging to the same genetic cluster, were sequenced using the Ion PGM™ Hi-Q™ Kit and the Ion 316™ Chip v2. Differences were detected in the microbiome compositions between *Ctenocephalides felis*, *Ctenocephalides canis* and *Ctenocephalides connatus.* Although based on a small sample, *C. connatus* occurring on wildlife harboured a higher bacterial richness when compared to *C. felis* on domestic animals. Intraspecific differences in the microbial OTU diversity were detected within *C. f. felis* that occurred on domestic cats and dogs. Different genetic lineages of *C. f. felis* were similar in microbial compositions but some differences exist in the presence or absence of rare bacteria. *Rickettsia* and *Bartonella* OTU's identified in South African cat fleas differ from those identified in the USA and Australia. Intraspecific microbial compositions also differ across geographic sampling sites. Generalized dissimilarity modelling showed that temperature and humidity are potentially important environmental factors explaining the pattern obtained.

## Graphical abstract

**Figure:**
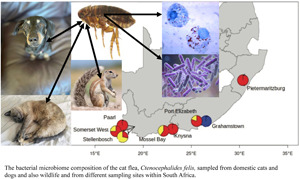

## Introduction

The genus *Ctenocephalides* comprises several species, which include the cat flea, *C. felis.* This well-known species is a haematophagous parasite of domestic cats and dogs and is of medical importance due to its close association with humans. With a nearly world-wide distribution, it is a known vector for various pathogenic microbes such as *Rickettsia*, and *Bartonella* (Bitam *et al*., 2010; Vasconcelos *et al*., 2018; Douglas *et al*., 2021; Manvell *et al*., 2022). Apart from zoonotic pathogens, ectoparasites also host a large diversity of non-pathogenic microbes (Vasconcelos *et al*., 2018). These non-pathogenic microbes can play an essential role in human and animal health as it has been reported that the overall microbial diversity can influence the acquisition, transmission and virulence of pathogens (Lawrence *et al*., 2015; Bonnet *et al*., 2017; Manvell *et al*., 2022). Despite the medical importance of the bacteria associated with *C. felis* (Manvell *et al*., 2022), contemporary insights into the microbiome of this taxon are limited to a few studies documenting pathogenic and non-pathogenic microbes (Lawrence *et al*., 2015; Vasconcelos *et al*., 2018; Douglas *et al*., 2021; Manvell *et al*., 2022). It is important to realize that the accurate detection of microbiota is based on many assumptions and pitfalls (Kim *et al*., 2017) and more accurate data can be generated when results are compared between studies done on similar host species, and from different geographic regions.

A study by Lawrence *et al*. (2015), documented the microbiome diversity of *C. felis* in Australia for the first time and showed species specific differences between the microbiomes of *C. f. felis,* and that of the closely related stickfast flea, *Echidnophaga a. ambulans.* Vasconcelos *et al*. (2018), studied *C. felis* from the United States of America (USA; northern and southern California) and confirmed that *Rickettsia*, *Bartonella* and the symbiotic *Wolbachia* were the most common bacteria in the cat flea. The same study showed that there was no overall difference in microbiome diversity between geographic regions but when the prominent *Rickettsia* reads were excluded, some geographic differentiation was detected with *Wolbachia* and *Bartonella,* being more abundant in fleas from southern California when compared to northern California. Most recently, Manvell *et al*. (2022) confirmed that the microbiome composition of *C. felis* differ among geographic sampling sites and concluded that widespread co-infection exist for strains of *Rickettsia*, *Bartonella* and *Wolbachia* across several states in the USA and the United Kingdom (UK). Although these studies provide some insights into the microbiome of the cat flea, the factors that drive the patterns in diversity and distribution are still to a large extent unknown.

At the broader scale, haematophagous obligate ectoparasites represent an interesting system to investigate the potential drivers of the variation in microbiome composition. The external factors that influence the microbiome of these parasite are likely more limited than, for example, free-living animals because of their specialized diets and their limited movement in the environment due to their host dependence. The microbial composition of these taxa is however part of the holobiont (characterized by a multidimensional interaction between the bacteria and the host, and also the many other species living in or around it; see also Ben-Yosef *et al*., 2017; McCabe *et al*., 2020; Doña *et al*., 2021) and as such several factors (e.g. parasite genetics, host ecology and evolution, abiotic environment, bacterial interactions and/or stochastic events) may act as potential drivers of a parasite's microbial community. Indeed, parasite identity was the strongest predictor of microbiome composition in bat flies while the environment also had an effect, albeit to a lesser degree (Speer *et al*., 2022). Similarly, Hawlena *et al*. (2013) found that the microbiomes of ectoparasites on a wild rodent were largely influenced by parasite identity (tick *vs* flea) but not by the host or environmental conditions across a small geographic scale. The authors attributed the unexplained variance in bacterial composition to interspecific bacterial interactions or stochastic events (Hawlena *et al*., 2013). Further, the study found no differences in the microbiome of 2 flea species (*Orchopeas leucopus* and *Ctenophthalmus pseudagyrtes*) when considering all bacterial phylotypes whereas differences were observed when considering only the commonest arthropod-specific genera (i.e. *Rickettsia*, *Franscisella* and *Bartonella*). The nature of variation in microbiomes and the relative importance of factors shaping this variation in ectoparasites remain understudied.

This study aims to provide data on the microbiome of *Ctenocephalides* fleas in South Africa and to document the role of host species, species identity (parasite genetics), and the external environment on the bacterial diversity of cat fleas (*C. felis*). We used a selection of flea samples obtained from 3352 flea individuals occurring on 576 domestic cats and dogs and 10 wildlife species across South Africa (van der Mescht *et al*., 2021). The latter study confirmed the existence of 3 monophyletic flea species (*C. felis*, *C. canis* and *C. connatus*) and suggested that of the 3 currently recognized cat flea subspecies (*C. f. damarensis, C. f. strongylus* and *C. f. felis*) only *C. f. damarensis* morphotypes collected from wildlife (ground squirrels) may be regarded as a distinct taxonomic entity (i.e. *C. f. damarensis* and *C. f. strongylus* obtained from cats and dogs should be synonymized with *C. f. felis*) (van der Mescht *et al*., 2021). Within *C. f. felis,* however, 2 genetically distinct lineages were detected, 1 confined to the western xeric and warmer region, whereas the other lineage was found in the xeric warmer and more mesic cooler regions in South Africa. It was suggested that the off-host environment, particularly temperature and humidity, are important factors to consider in the evolution of *C. felis* (van der Mescht *et al*., 2021; also see Crkvencic and Šlapeta, 2019).

Using the flea samples obtained from the above study, we superficially investigated whether the 3 *Ctenocephalides* species (*C. felis*, *C. canis* and *C. connatus*) and the 2 cat flea subspecies, *C. f. damarensis* (occurring on wildlife), and *C. f. felis* (occurring on cats and dogs) differ in their microbiome composition. Given the medical importance of the bacteria associated with *C. f. felis,* we more thoroughly investigated whether different vertebrate hosts (cats or dogs) have different microbial compositions and we also investigated whether the 2 genetic lineages of *C. f. felis* show different microbial species compositions. Since the environment was also shown to influence the microbiome of *C. f. felis* we explored which of selected environmental conditions will most likely affect the intraspecific microbial composition of *C. f. felis* geographically.

## Materials and methods

### Sampling

**Figure 1.**
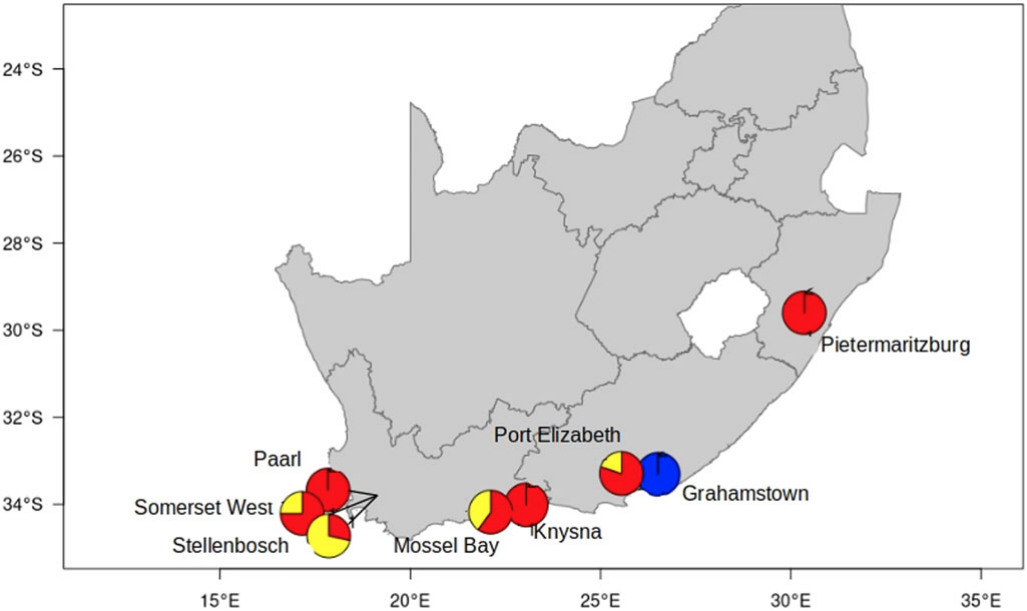
Sampling locations of *Ctenocephalides felis* used in the present study. Haplogroups as delineated by COII sequencing of flea samples (van der Mescht *et al*., 2021) and represented by haplogroups C1 = red, C2 = yellow and C3 = blue.

A total of 107 female fleas were selected from 8 distinct sampling sites in South Africa and included 3 *Ctenocephalides* species (*C. felis*, *C. canis* and *C. connatus*), 2 genetically and morphologically distinct *C. felis* subspecies (*C. f. damarensis* and *C. f. felis*), and the 2 genetic clades (C1 & C2) of *C. f. felis* (van der Mescht *et al*., 2021; Figure 1, Table 1). Flea collection and animal handling were performed after obtaining the necessary permits from the Department of Agriculture, Land Reform and Rural Development of the Republic of South Africa [12/11/1/7/5 (960)] and permission from Stellenbosch University Animal Ethics Committee (|ACU-2018–8860 and ACU- 2019–9089).

**Table 1.** Flea samples used in the current study, selected from 8 distinct South African sampling sites (locality and pool number) and including 3 different *Ctenocephalides* species (*C. felis*, *C. canis* and *C. connatus*), 2 genetically and morphologically distinct *C. felis* subspecies (*C. f. damarensis* and *C. f. felis*), and the 2 genetic clades of *C. f. felis* originating from cats and from dogs respectively (Genetic clade) (van der Mescht *et al*., 2021)

Flea species/Sample number (# individuals in pool)	Host	Locality and pool number	Locality abbreviation	Genetic clade
***Ctenocephalides canis***	Dog			
D70 (3)		Stellenbosch8	Stb	-
D312 (3)		Knysna	Kn	-
D313 (3)		Knysna2	Kn	-
***Ctenocephalides connatus***	Ground_squirrel (GS)			
W1289 (3)		Bloemhof	Bl	-
W1225 (3)		Bloemhof2	Bl	-
***Ctenocephalides f. damarensis***	Gennet (Gen)			
W2 (3)		Grahamstown	Grh	C3
W3 (3)		Grahamstown2	Grh	C3
***Ctenocephalides f. felis***	Cat			
D102 (3)		Mossel_Bay3	MB	C2
D103 (3)		Mossel_Bay5	MB	C1
D112 (3)		Mossel_Bay	MB	C1
D114 (3)		Mossel_Bay4	MB	C2
D127 (2)		Mossel_Bay2	MB	C1
D241 (3)		Paarl3	Prl	C1
D242 (3)		Paarl	Prl	C1
D243 (3)		Paarl2	Prl	C1
D184 (3)		Pietermaritzburg5	Pmb	C1
D187 (3)		Pietermaritzburg4	Pmb	C1
D189 (3)		Pietermaritzburg3	Pmb	C1
D201 (1)		Pietermaritzburg7	Pmb	C1
D207 (1)		Pietermaritzburg8	Pmb	C1
D258 (3)		Port_Elizabeth3	PE	C1
D259 (3)		Port_Elizabeth2	PE	C1
D260 (3)		Port_Elizabeth5	PE	C1
D269 (3)		Port_Elizabeth	PE	C1
D270 (3)		Port_Elizabeth4	PE	C1/C2
D166 (3)		Somerset_West2	SW	C2
D169 (3)		Somerset_West3	SW	C1
D171 (3)		Somerset_West	SW	C1
D172 (2)		Somerset_West4	SW	C1
D154 (3)		Stellenbosch2	Stb	C2
D19 (2)		Stellenbosch7	Stb	C2
D22 (2)		Stellenbosch6	Stb	C2
D45 (2)		Stellenbosch3	Stb	C2
D72 (3)		Stellenbosch5	Stb	C2
***Ctenocephalides f. felis***	Dog			
D140 (2)		Pietermaritzburg6	Pmb	C1
D143 (3)		Pietermaritzburg	Pmb	C2
D145 (3)		Pietermaritzburg2	Pmb	C2
D16 (3)		Stellenbosch	Stb	C1 + C2
D31 (3)		Stellenbosch4	Stb	C1

Fleas were collected using forceps and each individual was placed in a separate plastic tube filled with 96% ethanol and stored at ~ 5^◦^ C until further processing. Before DNA extraction, fleas were identified to species level using a Leica stereoscopic microscope (Leica Microsystems, Wetzlar, Germany) and the taxonomic key of Segerman (1995), followed by identification to subspecies and clades using morphological and genetic barcoding analyses (van der Mescht *et al*., 2021). The latter was done by amplification of the mitochondrial cytochrome c oxidase subunit II (COII) as described in van der Mescht *et al*. (2021).

### DNA extraction and sequencing

To eliminate surface contamination, each individual flea was washed in 3 solutions consecutively (1% sodium hypochlorite – NaClO; Phosphate-buffered saline – PBS, pH = 7.4; Absolute ethanol – 100% EtOH) followed by 3 sequential rinses in PBS. Each wash/rinse was done using 400 *μ*l of solution in a 1.5 mL tube for 1 min with gentle agitation. Genomic DNA isolation was performed in a DNA free environment and proceeded by crushing individual air-dried flea individuals in a 1.5 mL tube with 80 *μ*l of the NucleoSpin^®^ Tissue lysis buffer (Macherey-Nagel) for 3 min. The latter procedure was conducted using a bleached sterilized autoclaved plastic pestle (Eppendorf from Sigma Aldrich). After this homogenization step, 100 *μ*l lysis buffer and 25 *μ*l Proteinase K (Qiagen) was added to the solution. Complete genomic DNA isolation was then conducted using NucleoSpin® Tissue kit (Macherey-Nagel) following the instructions from the manufacturer. DNA was eluted in 50 *μ*l Tris buffer (pH = 8.5) and stored at −20°C until further processing.

Prior to sequencing, extracted DNA was quantified using Qubit (Life Technologies) assessment. DNA extracted from 2 to 3 fleas obtained from the same host sampled at the same site, and representing the same genetic cluster (Table 1) were pooled together and concentrations of DNA adjusted so that each individual's DNA was equally represented in the pooled sample. These samples were then amplified using standard procedures outlined in the Ion 16S™ Metagenomics Kit (Thermo Fisher Scientific Inc.) that contains 2 16S rRNA gene primer sets (Primer set V2-4-8 and Primer set V3-6, 7-9) that selectively amplify the corresponding hypervariable gene regions of the 16S rRNA of bacteria. A non-template DNA extraction blank was incorporated as a negative control (all chemicals and procedures used for the DNA extraction were utilized except no extracted flea DNA was added as part of the extraction step). A total of 39 pooled DNA samples (Table 1) and the 1 negative control were subsequently sequenced at the Central Analytical Facility of Stellenbosch University using the Ion PGM™ Hi-Q™ Sequencing Kit and the Ion 316™ Chip v2 (Thermo Fisher Scientific Inc.). The data were generated on an GeneStudio S5 Prime System (Ion Torrent PGM sequencer; Thermo Fisher Scientific Inc.) and the 530 Chip was set to 850 flows that was sufficient to generate 400 bp fragments.

### Data analyses

The Torrent Suite v4.4.2 (Thermo Fisher Scientific Inc.) was used for base calling and demultiplexing of sequencing reads using default parameters. Sequences were processed for quality and initial analyses performed using Ion Reporter software v5.10 (Thermo Fisher Scientific Inc.) with cloud-based software and default settings (Malczynski *et al*., 2021). The Ion Reporter Software uses a curated 16S rRNA gene reference database (MicroSEQ ID) as well as the curated Greengenes database (McDonald *et al*., 2012) for annotation of microbial sequences. Reads filtered for quality (>Q20) were used to pick operational taxonomic units (OTUs) utilizing an open-reference OTU picking method based on 97% identity to sequences in the Greengenes database (v13.5) as per the manufacturer's instructions. This Ion Reporter Metagenomic Workflow (Thermo Fisher Scientific Inc.) generates read counts, percentage mapped reads (including percentage mapped reads per primer), percentage sequence match and information on mapping. The criteria for taxonomic calls by the Ion Reporter metagenomic workflow are as follows: family, genus or species level identification are accepted when the read count is greater or equal to 1000; the percentage of mapped reads per primer is equal to or greater than 25% and the percentage of sequence match is equal to or greater than 97%.

Prior to statistical analysis, all OTUs with counts lower than 10 were removed from the dataset. In addition, OTUs identified in the negative control were also removed from the dataset entirely as well as OTUs not present in 10% of *C. f. felis* or *C. canis* samples or 100% of *C. damarensis* or *C. connatus* samples. All statistical analyses of the dataset were conducted using R (R version 4.1.3 (2022-03-10)) and total OTU counts. Firstly, community (alpha) diversity assessment consisting of observed richness, Shannon and Simpson diversity indexes and relative abundance, was conducted through processing of OTU counts using the R package phyloseq (McMurdie and Holmes, 2013). The phyloseq function, phyloseq::rerfy_even_depth was used to sub-sample the data without replacement in order to normalize OTU counts resulting from different sample sizes. Statistical significance of differences in relative abundance between flea species and between host species was calculated as per La Rosa *et al*. (2012). The latter assumes a Dirichlet-Multinomial distribution and testing for a difference in mean distribution of each taxon across groups is conducted, accounting for the overdispersion in count data. Rare taxa (with counts under 100) are pooled into 1 group. Statistical significance of differences in observed richness and Shannon and Simpson diversity indexes between flea species and between host species was calculated using a Wilcoxon rank-sum exact test. Next, OTU counts at the genus level were displayed graphically. All graphics were generated using R package *ggplot2* (Wickham 2016) unless stated otherwise. The genus level taxonomic rank was selected to highlight the counts for *Ricketssia, Wolbachia* and *Bartonella* which form the focus of this study. Bacterial community differences for *C. felis* samples from felines and canines were visualized using Bray–Curtis dissimilarity and a non-metric multidimensional scaling (NMDS) plot, drawn using the R package *vegan* (Oksanen *et al*., 2012) and based on sequence counts of OTUs. To investigate possible geographic structure among the most common pathogenic bacteria found, phylogenetic analyses of *Rickettsia* and *Bartonella* found in *C. f. felis* sampled from cats only were performed. Ten random taxon input sequences were used for parsimony and maximum likelihood searches in PAUP* 4.0a169 (Swofford, 2021). Midpoint rooting was used and nodal support was estimated using 1000 bootstrap replicates. The optimal model for sequence evolution was determined using the Automated model selection function in PAUP* 4.0a169 (Swofford, 2021).

Generalized dissimilarity modelling (GDM; Ferrier *et al*., 2002, 2007) was used to record the environmental factors that potentially drives species turnover in the *C. felis* microbiome [i.e. change of pairwise dissimilarity (i.e. beta-diversity) along environmental gradients]. The GDM is a non-linear extension of the matrix regression technique. Its main advantage for studying the spatial patterns of species turnover is that it takes into account 2 types of non-linearity inherent to ecological data (Ferrier *et al*., 2007), namely (a) variable rate of compositional turnover along environmental gradient(s) and (b) the fact that the relationship between pairwise between-site compositional dissimilarity and environmental/spatial gradient(s) is curvilinear rather than linear (Ferrier *et al*., 2007). Consequently, the GDM deals with the variation of the turnover rate along each gradient by transformation of each of the predictor variables using an iterative maximum-likelihood estimation and I-splines (Ferrier *et al*., 2007; Fitzpatrick *et al*., 2013). The maximum height of each plotted I-spline indicates the total amount of turnover associated with a given gradient, while all other predictors are held constant, so that the I-splines represent partial regression fits, demonstrating the importance of the effect of each predictor on compositional turnover. The slope of the I-spline demonstrated the turnover rate and its variation along a given gradient. To account for the curvilinear relationship between dissimilarity and environmental/spatial gradient(s), the linear predictor variable is transformed *via* a link function that defines the relationship between pairwise between-site dissimilarities (constrained to the range from zero to unity) and a scaled combination of between-site distances based on any number of environmental or geographical variables (see details in Ferrier *et al*., 2007). To run the GDMs, we used the package ‘gdm’ (Fitzpatrick *et al*., 2022) implemented in R. Data were transformed to presence/absence of species present at each geographic sampling locality. Data on environmental variables that presumably affect physiological processes in fleas (see Krasnov, 2008) were extracted from CHELSA database for January 2019 (Karger *et al*., 2017; Brun *et al*., 2022). These variables were climate moisture index (kg/m^2^ per month), near surface relative humidity (%), potential evapotranspiration (kg/m^2^ per month), precipitation amount (kg/m^2^ per month/100), mean daily air temperature (°) and vapour pressure deficit (Pa) as outlined to be important in Krasnov (2008).

## Results

### Microbiome diversity among and within flea species

**Figure 2.**
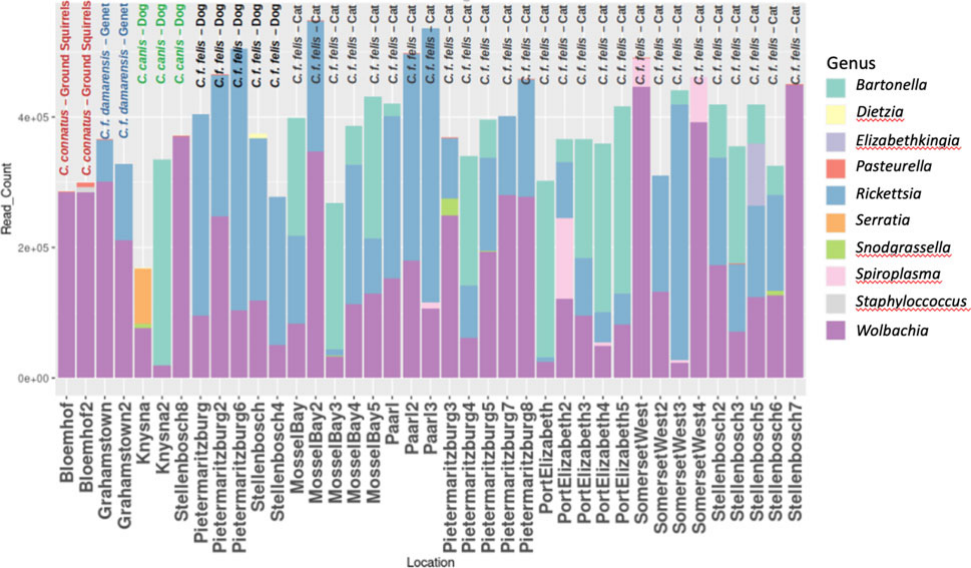
Read counts for the 10 most common genera found across the 39 pooled *Ctenocephalides* samples sequenced in the present study. *Ctenocephalides* species and subspecies, together with the common name of the hosts from where they were sampled are indicated. Locality names correspond to those in Table 1.

**Figure 3.**
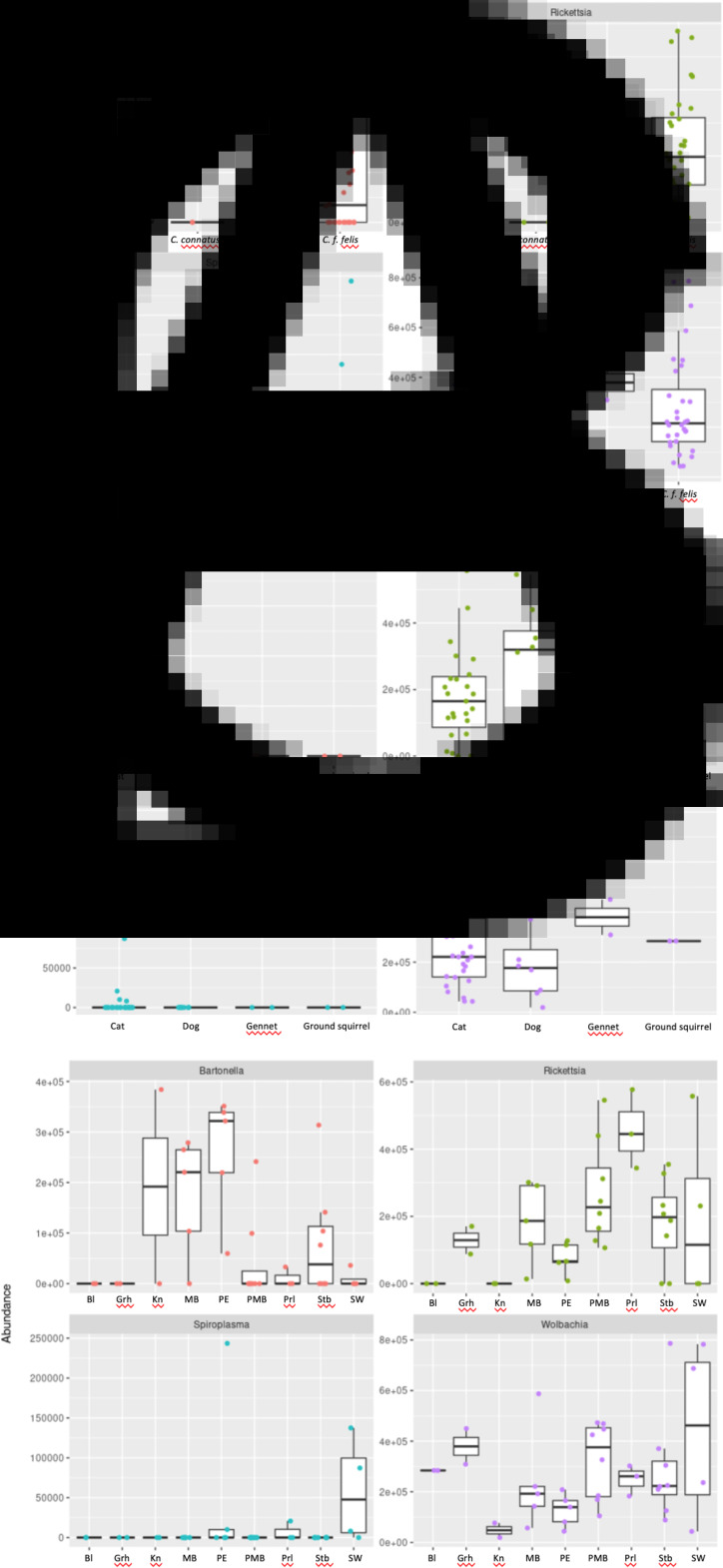
Relative abundance for the OTU counts greater than 100 000 found across the 39 pooled *Ctenocephalides* samples sequenced in the present study depicting the most common genera found in the dataset. (A) Relative abundance across flea species, (B) Relative abundance across host species, (C) Relative abundance per sampled locality. Locality abbreviations and host abbreviations correspond to those in Table 1.

The 107 *Ctenocephalides* samples sequenced in 39 pools yielded 453 168 to 893 406 reads per sample which passed quality filtering, and a total 387 OTUs belonging to 91 families, 116 genera and 180 putative species were identified. All data are available under Bioproject accession number PRJNA1007429. After comparison to the negative control, 20 families, 23 genera and 19 species were omitted. Furthermore, all OTUs not present in at least 10% of *C. f. felis* or *C. canis* or 100% of *C. connatus* or *C. damarensis* samples, were removed from the dataset. The remaining OTUs consisted of 25 families, 30 genera and 37 species. The most abundant bacterial genera (as per OTU counts summed across samples) found in the 3 *Ctenocephalides* species studied herein were *Spiroplasma*, *Rickettsia, Wolbachia* and *Bartonella* (Fig. 2). Variable levels of relative abundance was detected for *Spiroplasma, Rickettsia, Wolbachia* and *Bartonella* among the flea species and OTU counts were generally greatest in *C. felis* followed by *C. canis* and *C. damarensis* (the latter for *Rickettsia* and *Wolbachia* only) (Fig. 3A). These differences in abundance may however be strongly influenced by the large differences in sample numbers between *C. felis* and the other flea species. The differences in abundance of all bacterial genera between *C. felis* and *C. canis* are not significant (P = 1, 12 df) (Fig. 3A) and neither is the difference in abundance between *C. felis* and *C. damarensis* (P = 0.19, 12 df) (Fig. 3A). Relative abundance of the same common bacteria was highest in feline hosts for *Bartonella* and *Spiroplasma,* canine hosts for *Rickettsia* and Gennets for *Wolbachia* (Fig. 3B). The difference in abundance of all bacterial genera found in *C. f. felis* between canine and feline hosts is significant (P = 0.03, 12 df) with a total of 16 genera unique to *C. f. felis* samples from cats, 1 genus unique to *C. f. felis* sampled from dogs, and 6 genera shared among the fleas sampled from these 2 hosts (Supplementary Table 1), but the difference between *C. f. felis* on feline hosts and Gennets is not significant (P = 0.07, 12 df) and neither is the difference in relative abundance between *C. felis* and canine hosts and Gennets (P = 0.8, 12 df) (Fig. 3B). Relative abundance across sampling locations (Fig. 3C) was highest for *Bartonella* in Port Elizabeth, *Rickettsia* in Paarl, *Spiroplasma* in Somerset West and *Wolbachia* in Somerset West (Fig. 3C). In flea taxa where sampling was limited, *Rickettsia* species were not identified in any *C. connatus,* with only Ricketsiaceae identified and the *Rickettsia* genus at very low counts. This was also the case in *C. canis*. Host differences were also detected in that *Bartonella* were absent in *C. f. felis* samples obtained from dogs, but at the family level, *Bartonellaceae* was found at low frequency in 1 pooled sample (Supplementary Table 1).

The different flea species sampled in the present study also showed variable bacterial profiles (Supplementary Table 1). *Ctenocephalides canis*, *C. f. damarensis* and *C. f. felis* from canine hosts had no unique bacterial taxa while *C. connatus* had 7 unique bacteria taxa which included, among others, *Staphylococcus succinus, Streptomyces* and an unclassified *Rhizobiales* (Supplementary Table 1). *Ctenocephalides f. felis* from feline hosts had the greatest number of unique bacterial taxa which based on sequence similarity and BLASTn searches (>97% sequence identity) included OTU's closely related to *Bartonella grahamii* and *B. henselae* as well as *Rickettsia sibrica* (Supplementary Table 1).

The NMDS plot of differences in bacterial communities between *C. f. felis* samples from canines and felines measured using Bray–Curtis dissimilarity show the rank-order distances between points. The greater the distance between points on the plot, the greater the difference in bacterial community makeup. There is overall greater difference between bacterial communities from feline hosts as they show a greater spread on the plot (Supplementary Fig. 1). Bacterial communities from canine hosts show fewer or smaller differences between samples as they are clustered more closely together on the plot. In addition, bacterial communities from canine hosts also cluster more closely with some samples from feline hosts, indicating smaller differences between those specific bacterial communities. These differences in bacterial community composition is reflective of the differences in bacterial abundance observed between canine and feline hosts and that samples from felines are more different from one another than samples from canines (Supplementary Fig. 1).

**Figure 4.**
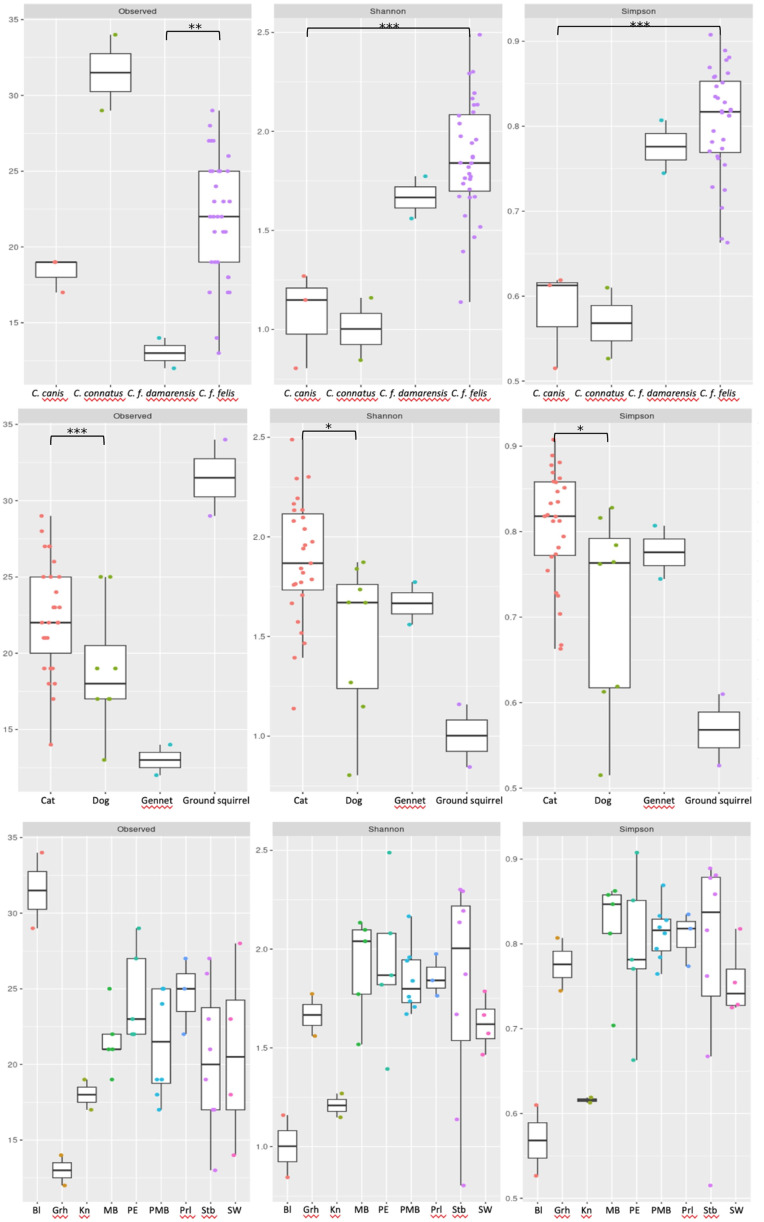
Observed richness, Shannon and Simpson diversity indexes across (A) flea species, (B) host species where GS = Ground Squirrel; G = Gennet and (C) locations as listed in Table 1.

Alpha-diversity metrics including observed richness, Shannon diversity index and Simpson diversity index was highest in *C. felis* in all cases except for observed richness which was higher in *C. conatus* (Fig. 4A). However, this may again be influenced by the smaller sample sizes for some species. Nonetheless, the Wilcoxon rank-sum test comparing canine to feline hosts showed significant differences in observed richness (P = 0.03), and Shannon and Simpson diversity indexes (P = 0.015 and 0.017 respectively). There is no significant difference in the observed richness estimates across *C. felis* and *C. canis* (P = 0.1) but there is significant differences in Shannon and Simpson diversity estimates between *C. felis* and *C. canis* (P = 0.001 and P = 0.0003, respectively). A similar trend was, however, seen in that all indices were highest for feline hosts except for observed richness which was higher in Ground Squirrels (albeit based on small sample size) (Fig. 4B). This trend is followed in the observed richness being highest in the Bloemhof location, from which only 2 pooled samples were obtained. The Stellenbosch location had the highest diversity indexes (Fig. 4C). Comparing observed richness and Shannon and Simpson diversity estimates across *C. felis* and *C. damarensis* flea species showed significant difference for observed richness (P = 0.02) but there is no significant differences in Shannon and Simpson diversity estimates (P = 0.3 in both instances).

### Geographic structure in the OTU diversity of the cat flea microbiome

The common bacteria present in *C. felis* revealed no clear geographic differences in alpha-diversity metrics (Fig. 4C) and Wilcoxon rank-sum tests confirmed no statistical significant differences (0.2 < P < 0.9). Phylogenetic analyses of the *Rickettsia* and *Bartonella* OTU's detected in *C. f. felis* in this study only revealed the presence of several distinct 16S rRNA lineages for South African cat fleas but there were no clear geographic patterns among the sampling sites nor any genetic association specific to vertebrate host taxa (data not shown). Concerning *Bartonella,* 1 taxon did not cluster with any of the known species on Genbank and 6 different OTU clusters were detected in *C. f. felis* on cats in South Africa. Based on the total number of sequences obtained across all samples, the most common OTU's detected in this study were mostly similar to *B. clarridgeiae* and *B. henselae* on Genbank followed by sequences similar to *B. grahamii*. The remaining 3 OTU clusters share >97% sequence similarity with *B. quintana, B. rattaustraliani,* and *B. rochalimae* but these were detected in very low frequencies. Fifteen *Rickettsia* OTU's were observed. Of these, the most common OTU clusters found in this study most closely represent *R. australis, R. rickettsia,* and *R. hoogstraalii* in Genbank (Supplementary Table 1).

### Environmental factors that may affect the microbiome composition of *C. f. felis*

**Figure 5.**
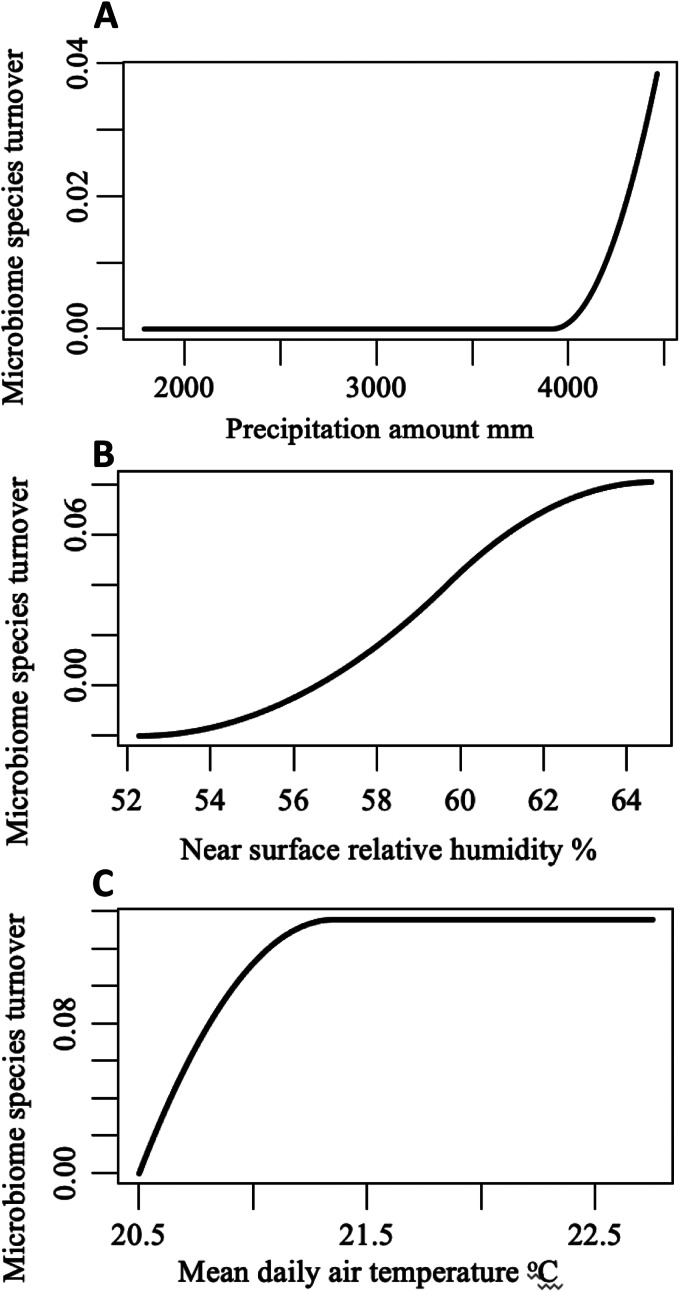
Generalized dissimilarity model-fitted I-splines (partial regression fits) of (A) amount of precipitation in mm, (B) near surface relative humidity in percentage, and (C) mean daily air temperature in degrees centigrade as predictors of *C. f. felis* microbiome species turnover. The steeper slope of the transformed relationship on a given section of the gradient indicates greater rate of a turnover.

Beta diversity of the microbiome composition at each site revealed that the percentage of explained deviance in the GDM was relatively low (19.24%). Nevertheless, the GDM revealed the main drivers of microbiome species turnover across sites being daily mean temperature, near-surface humidity and, to a lesser extent, precipitation (Table 2, Fig. 5). Pairwise dissimilarity in microbiome species composition steadily increased along the gradient of near surface relative humidity (Fig. 5). Along most of the precipitation gradient, this dissimilarity was extremely low and then sharply increased at the mesic sites. In contrast, the rate of species turnover was the highest at the colder sites and then slowed down (Fig. 5).

**Table 2.** Coefficients of the I-splines produced by the GDM of relationships between species turnover in the microbiome of *C. f. felis* sampled from cats only and across localities and geographic distance and climatic variables

Gradient	I-Spline			Sum of coefficients
	1	2	3	
Mean daily air temperature	0.136	0.00	0.00	0.136
Near surface relative humidity	0.00	0.101	0.00	0.101
Precipitation amount	0.00	0.00	0.04	0.04
Geographic distance	0.00	0.02	0.00	0.02
Climate moisture index	0.00	0.00	0.00	0.00
Potential evapotranspiration	0.00	0.00	0.00	0.00
Vapour pressure deficit	0.00	0.00	0.00	0.00

## Discussion

The present study, mainly documenting bacterial diversity in *C. felis* fleas confined to South African host taxa, confirm the notion that host identity, parasite identity (flea species), and climatic conditions influence the microbiome compositions (Lawrence *et al*., 2015; Vecchi *et al*., 2018; Storo *et al*., 2021). Our study confirm that *Bartonella, Rickettsia* and *Wolbachia* are the most prevalent bacteria present in *C. f. felis* from cats but *Bartonella* was absent in *C. f. felis* sampled from dogs. Further, closely related subspecies of the cat flea, *C. f. felis* (on cats and dogs) and *C. f. damarensis* (from wildlife), showed significant differences in species richness (based on small sample sizes). Although these differences may be attributed to the relevant vertebrate host species, the influence of the environment off the host was not thoroughly investigated and thus cannot be excluded as a contributing factor explaining the pattern obtained (Griffiths *et al*., 2019; Cahana and Iraqi, 2020). The genetically distinct *C. f. felis* clades, however, sampled from the same host species (van der Mescht *et al*., 2021) show broadly similar microbiome compositions (but differ in rare OTU's; also see Supplementary Fig. 1), and some differences are detected in beta-diversity among sampling sites.

When host species differences are eliminated as a contributing factor to microbiome diversity, interesting large-scale geographic differences were detected between the pathogenic microbiomes of *C. f. felis* from South African cats when compared to a similar study in California, USA (Vasconcelos *et al*., 2018). In the case of *Rickettsia,* a bacterium genus that is responsible for febrile illness globally, *R. felis* was the most abundant in the USA study followed by *R. senegalensis* (Vasconcelos *et al*., 2018). In South African samples, sequences obtained were most similar to *R. australis* which represent the most prominent lineage, followed by 2 OTU groups representing sequences most similar to *R. felis* and *R. hoogstraalii* (found in a lower number of amplicons). Despite detecting 15 different *Rickettsia* OTU lineages in the present study, the most common *R. senegalensis* detected in the USA, did not cluster close to any of the OTU lineages in the present study. In the case of *Bartonella,* that is associated with cat scratch disease, the presence of both *B. henselae* and *B. clarridgeiae* like sequences in the present study support the observations in California, USA. However, *B. koehlerae,* mostly confined to the northern sites in California, was absent from the South African fleas. The fourth most abundant bacterial species in the Californian study, *Snodgrassella alvi*, is a bacterium that is commonly associated with the gut microbiome of honey bees (*Apis* spp.) and bumble bees (*Bombus* spp.) (Kwong *et al*., 2014), and it was also found in the gut microbiome of Australian cat fleas (Lawrence *et al*., 2015). Interestingly, none of the sequences obtained in the present study cluster closely with *S. alvi* and this suggest that the latter taxon is most likely absent in South African cat fleas. *Spiroplasma*, a bacteria that is associated with intraocular infections in humans (Matet *et al*., 2020) was the fourth most prominent taxon detected in South Africa followed by *Elizabethkingia* which is documented as a multi-drug-resistant bacteria that can be associated with urinary tract infection (Gupta *et al*., 2017). These findings confirm the notion that the external environment (e.g. habitat, geography) can influence the microbiome composition (Ahn and Hayes, 2021; Kapheim, Johnson and Jolley, 2021).

It has also been postulated that host ecology can influence the microbiome diversity (Lawrence *et al*., 2015). In the present study we compared the microbiome on congeneric flea species (*C. connatus*, *C. canis* and *C. felis*), that vary in host preference. The bacterial OTU richness was markedly higher and more unique in *C. connatus* that is associated with natural occurring burrowing rodents (ground squirrels) when compared to *C. canis* and *C. felis* that occur on domestic cats and dogs. In fact at least 2 of the unique bacteria in *C. nonnatus* are associated with soil (*Ralstonia* spp.; Zhang *et al*., 2020) and plant roots (*Methylobacterium* spp.; Palberg *et al*., 2022). This pattern is supported by Lawrence *et al*. (2015) that recorded a higher bacterial richness in the stickfast flea, *E. a. ambulans*, that occur on echidnas (another burrowing host species; Morrow and Nicol 2009) compared to the gut microbiome of cat fleas, *C. f. felis*. It is probable that adult fleas (on the host body) and the free-living pre-imago life stages (in the nest) of burrowing host species will be more exposed to soil and its diverse bacterial composition (Torsvik *et al*., 1990) and this will facilitate a higher bacterial richness in these species when compared to cat fleas, whose domestic hosts generally live either indoors or in a relatively constant and confined outdoor environment (Lawrence *et al*., 2015).

Environmental conditions, and specifically macroclimatic conditions can play a major role in the microbiome diversity of parasites (Wielinga *et al*., 2006; Clay *et al*., 2008). The present study supports this through the GDM results. Dissimilarity in microbiome species composition was mainly driven by among-site variation in daily mean temperature, near-surface humidity and precipitation, although the relationship with the latter factor was weak. Since mean annual temperature and mean annual precipitation has also been implicated as the main driver for bacterial community dissimilarities in forests (Liu *et al*., 2022), the environment off the host is clearly an important factor to consider. It is, however, interesting to note that temperature and humidity has also been proposed to affect flea distribution and abundance since the immature stages of fleas are sensitive to desiccation when they develop in the nest of the host (Kreppel *et al*., 2016; van der Mescht *et al*., 2021). Based on the data at hand, it may be a mere co-incidence that flea and microbiome diversity are influenced by the same climatic factors (temperature and humidity), but on the other hand it may also reflect the close relationship between the evolution of the host (flea in this instance) and the bacteria associated with it.

## Supporting information

Matthee et al. supplementary material 1 Matthee et al. supplementary material

Matthee et al. supplementary material 2 Matthee et al. supplementary material

## Data Availability

All data are available under Bioproject accession number PRJNA1007429.

## Supplementary material

The supplementary material for this article can be found at https://doi.org/10.1017/S0031182023000835
