# Supplementary material for: Documenting the microbiome diversity and distribution in selected fleas from South Africa with an emphasis on the cat flea, Ctenocephalides f. felis
Source: Parasitology. 2023 Sep 8;150(11):979–89. doi: 10.1017/S0031182023000835 (PMC10941216; doi:10.1017/S0031182023000835)
Supplement: Matthee et al. supplementary material 1 — Matthee et al. supplementary material [file S0031182023000835sup001.docx]

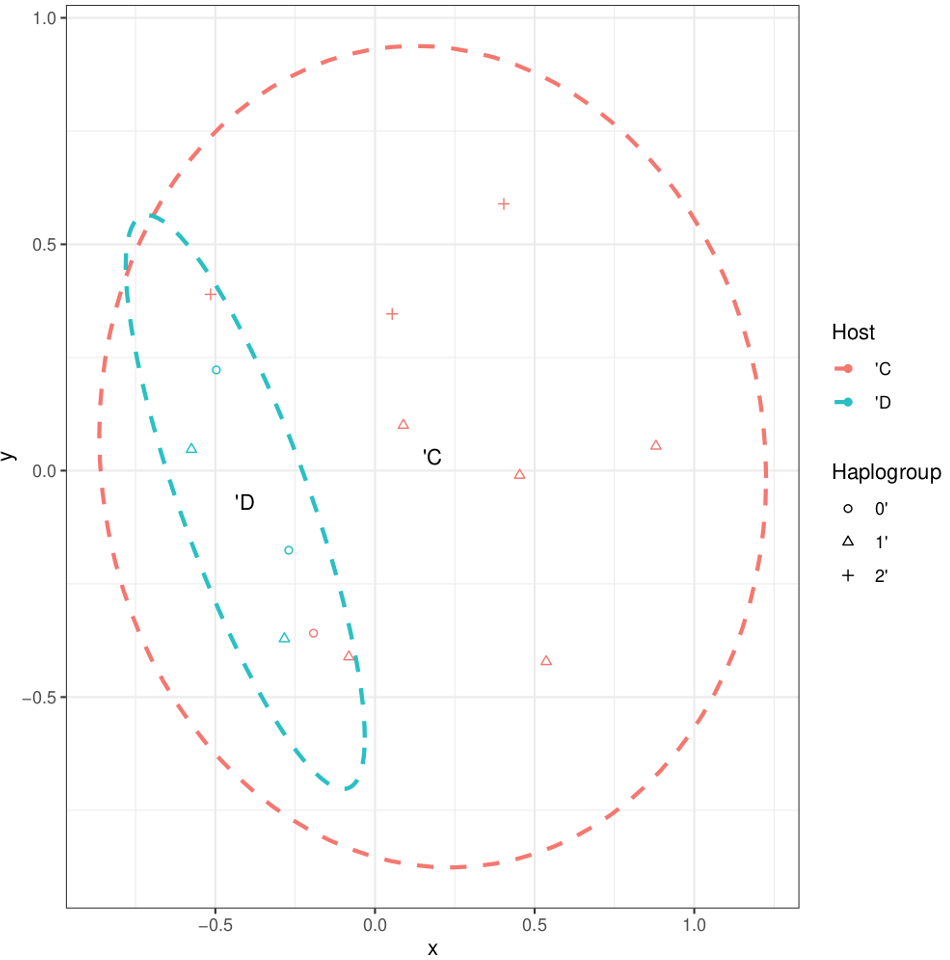


Supplementary Figure 1: Non-metric multidimensional scaling plot of bacterial composition discerned using Bray-Curtis dissimilarity between *C. f. felis* samples from Canine (D) and Feline (C) vertebrate hosts. Haplogroups correspond to genetic assemblages as described in Table 1
