# Supplementary material for: Documenting the microbiome diversity and distribution in selected fleas from South Africa with an emphasis on the cat flea, Ctenocephalides f. felis
Source: Parasitology. 2023 Sep 8;150(11):979–89. doi: 10.1017/S0031182023000835 (PMC10941216; doi:10.1017/S0031182023000835)
Supplement: Matthee et al. supplementary material 2 — Matthee et al. supplementary material [file S0031182023000835sup002.docx]

**Supplementary Table 1:** *Ctenocephalides* species and subspecies sampled in the present study indicating the bacterial taxa (f, family; g, genus and s, species) unique only to one *Ctenocephalides* species or subspecies in each instance. Species names listed herein are approximate and not certain since they are defined based on a short fragment of 16S rRNA and based on similarity scores only. Values in the columns indicate the percentage of samples that the bacterial taxon was identified in.

| **Bacterial family/genus/species** | ***Ctenocephalides f. felis* (Cat)** | ***Ctenocephalides f. felis* (Dog)** | ***Ctenocephalides canis*** | ***Ctenocephalides connatus*** | ***Ctenocephalides* *f.* *damarensis*** |
| --- | --- | --- | --- | --- | --- |
| *Arthrobacter_mysorens* (g,s) | -- | -- | -- | Y | -- |
| *Bartonella_grahamii* (g,s) | 32% | -- | -- | -- | -- |
| *Bartonella_henselae* (g,s) | 39% | -- | -- | -- | -- |
| *Brachybacterium_sp.* (g,s) | -- | -- | -- | 100% | -- |
| *Corynebacterium_variabile* (g,s) | 10% | -- | -- | -- | -- |
| Dermabacteraceae (f) | -- | -- | -- | 100% | -- |
| Dermacoccaceae (f) | -- | -- | -- | 100% | -- |
| *Ensifer_mexicanus* (g,s) | 14% | -- | -- | -- | -- |
| *Enterobacter_hormaechei* (g,s) | 7% | -- | -- | -- | -- |
| Gordoniaceae (f) | 17% | -- | -- | -- | -- |
| *Mannheimia_glucosida* (g,s) | -- | *--* | *--* | 100% | *--* |
| *Meiothermus* (g) | 7% | *--* | *--* | -- | *--* |
| Porphyromonadaceae (f) | 14% | -- | -- | -- | -- |
| *Porphyromonas* (g) | 7% | *--* | *--* | -- | *--* |
| Prevotellaceae (f) | 10% | -- | -- | -- | -- |
| *Prevotella* (g) | 10% | *--* | *--* | -- | *--* |
| Rhizobiaceae (f) | 46% | -- | -- | -- | -- |
| *Rhizobiales (unclassified)* (g) | -- | *--* | *--* | 100% | *--* |
| *Rickettsia_sibirica* (g,s) | 14% | *--* | *--* | -- | *--* |
| *Sinorhizobium* (g) | 10% | *--* | *--* | -- | *--* |
| Spiroplasmataceae (f) | 21% | -- | -- | -- | -- |
| *Spiroplasma* (g) | 21% | *--* | *--* | -- | *--* |
| *Spiroplasma_ixodetis* (g,s) | 21% | *--* | *--* | -- | *--* |
| *Spiroplasma_sp.* (g,s) | 21% | *--* | *--* | -- | *--* |
| *Staphylococcus_succinus* (g,s) | -- | *--* | *--* | 100% | *--* |
| *Streptomyces* (g) | -- | *--* | *--* | 100% | *--* |
| Thermaceae (f) | 7% | -- | -- | -- | -- |

**Supplementary Table 2:** Bacterium species detected in *C. f. felis* samples from cats and from 6 localities in South Africa. Species names listed herein are approximate and not certain since they are defined based on a short fragment of 16S rRNA and based on similarity scores only. The total number of reads for each bacterium is also provided

| **Taxon** | **Paarl** | **Mossel Bay** | **Port Elizabeth** | **Somerset West** | **Stellenbosch** | **Pietermaritzburg** | **Total** |
| --- | --- | --- | --- | --- | --- | --- | --- |
| ***Ruminococcus sp.*** | 0 | 0 | 0 | 0 | 0 | 1 | **13** |
| ***Chryseobacterium antarcticum*** | 0 | 0 | 0 | 0 | 1 | 0 | **41** |
| ***Staphylococcus hominis*** | 0 | 0 | 0 | 1 | 0 | 0 | **17** |
| ***Sphingomonas echinoides*** | 1 | 0 | 1 | 1 | 1 | 1 | **520** |
| ***Shewanella xiamenensis*** | 0 | 0 | 0 | 1 | 0 | 0 | **25** |
| ***Enterococcus faecalis*** | 0 | 0 | 0 | 0 | 1 | 0 | **14** |
| ***Acinetobacter baumannii*** | 0 | 0 | 0 | 0 | 1 | 0 | **24** |
| ***Rickettsia montanensis*** | 1 | 1 | 1 | 1 | 0 | 1 | **1013** |
| ***Klebsiella variicola*** | 0 | 0 | 0 | 0 | 1 | 0 | **18** |
| ***Rickettsia hoogstraalii*** | 1 | 1 | 1 | 1 | 1 | 1 | **180779** |
| ***Paenibacillus barcinonensis*** | 0 | 0 | 0 | 1 | 0 | 0 | **28** |
| ***Conchiformibius steedae*** | 0 | 0 | 0 | 0 | 0 | 1 | **12** |
| ***Rickettsia sp.*** | 1 | 1 | 0 | 1 | 0 | 0 | **87** |
| ***Sphingomonas glacialis*** | 1 | 0 | 0 | 0 | 0 | 0 | **11** |
| ***Rickettsia aeschlimannii*** | 1 | 1 | 0 | 0 | 0 | 0 | **32** |
| ***Caulobacter segnis*** | 0 | 0 | 0 | 0 | 1 | 0 | **12** |
| ***Conchiformibius kuhniae*** | 0 | 0 | 0 | 1 | 0 | 0 | **15** |
| ***Meiothermus cateniformans*** | 1 | 0 | 0 | 0 | 0 | 0 | **22** |
| ***Acinetobacter ursingii*** | 0 | 0 | 1 | 1 | 0 | 0 | **103** |
| ***Enterobacter hormaechei*** | 0 | 0 | 0 | 0 | 1 | 1 | **961** |
| ***Marmoricola scoriae*** | 0 | 0 | 0 | 0 | 0 | 1 | **33** |
| ***Bartonella rattaustraliani*** | 0 | 1 | 1 | 0 | 1 | 1 | **456** |
| ***Spiroplasma ixodetis*** | 1 | 0 | 1 | 1 | 0 | 0 | **135182** |
| ***Bartonella grahamii*** | 1 | 0 | 1 | 1 | 1 | 1 | **15859** |
| ***Rickettsia sibirica*** | 1 | 0 | 0 | 1 | 0 | 0 | **54** |
| ***Moraxella canis*** | 0 | 0 | 0 | 1 | 0 | 0 | **10** |
| ***Prevotella sp.*** | 0 | 0 | 0 | 1 | 0 | 0 | **34** |
| ***Ensifer mexicanus*** | 0 | 1 | 0 | 0 | 1 | 0 | **138** |
| ***Bartonella quintana*** | 0 | 1 | 1 | 0 | 0 | 1 | **103** |
| ***Elizabethkingia anophelis*** | 0 | 0 | 0 | 0 | 1 | 0 | **29** |
| ***Pseudomonas aeruginosa*** | 1 | 1 | 1 | 1 | 1 | 1 | **1375** |
| ***Ralstonia insidiosa*** | 1 | 1 | 1 | 1 | 1 | 1 | **4308** |
| ***Sphingobium herbicidovorans*** | 0 | 0 | 0 | 0 | 0 | 1 | **11** |
| ***Bartonella henselae*** | 1 | 1 | 1 | 1 | 1 | 1 | **422031** |
| ***Rickettsia australis*** | 1 | 1 | 1 | 1 | 1 | 1 | **997188** |
| ***Bartonella rochalimae*** | 0 | 1 | 1 | 0 | 0 | 1 | **450** |
| ***Methylobacterium zatmanii*** | 1 | 0 | 0 | 0 | 0 | 0 | **35** |
| ***Prevotella amnii*** | 0 | 0 | 1 | 0 | 0 | 0 | **55** |
| ***Pelomonas aquatica*** | 1 | 1 | 1 | 1 | 1 | 1 | **1391** |
| ***Tepidimonas taiwanensis*** | 0 | 0 | 0 | 1 | 0 | 0 | **11** |
| ***Corynebacterium thomssenii*** | 0 | 0 | 0 | 1 | 0 | 0 | **43** |
| ***Flectobacillus roseus*** | 0 | 0 | 0 | 1 | 0 | 0 | **13** |
| ***Corynebacterium kroppenstedtii*** | 0 | 0 | 1 | 0 | 0 | 0 | **227** |
| ***Rickettsia akari*** | 1 | 0 | 0 | 0 | 0 | 0 | **15** |
| ***Bartonella clarridgeiae*** | 0 | 1 | 1 | 0 | 0 | 1 | **406022** |
| ***Herbaspirillum rhizosphaerae*** | 0 | 0 | 0 | 1 | 0 | 0 | **11** |
| ***Pseudomonas psychrotolerans*** | 0 | 0 | 0 | 0 | 0 | 1 | **13** |
| ***Corynebacterium vitaeruminis*** | 0 | 0 | 0 | 0 | 1 | 0 | **35** |
| ***Rickettsia rickettsii*** | 1 | 1 | 1 | 1 | 1 | 1 | **2672** |
| ***Chryseobacterium hominis*** | 0 | 0 | 0 | 0 | 1 | 0 | **28** |
| ***Rickettsia asiatica*** | 1 | 0 | 0 | 0 | 0 | 0 | **59** |
| ***Gardnerella vaginalis*** | 0 | 0 | 1 | 0 | 0 | 0 | **51** |
| ***Porphyromonas gulae*** | 1 | 0 | 0 | 0 | 0 | 0 | **10** |
| ***Kocuria kristinae*** | 0 | 0 | 0 | 1 | 0 | 0 | **210** |
| ***Corynebacterium variabile*** | 0 | 0 | 0 | 0 | 1 | 1 | **76** |
| ***Arthrobacter russicus*** | 1 | 1 | 1 | 1 | 1 | 1 | **912** |
| ***Rickettsia massiliae*** | 1 | 1 | 1 | 1 | 0 | 1 | **260** |
| ***Streptococcus australis*** | 0 | 0 | 1 | 0 | 0 | 0 | **13** |
| ***Ralstonia mannitolilytica*** | 1 | 1 | 1 | 1 | 1 | 1 | **1722** |
| ***Pelomonas puraquae*** | 1 | 1 | 1 | 1 | 1 | 1 | **6607** |
| ***Rickettsia heilongjiangii*** | 1 | 1 | 0 | 1 | 1 | 1 | **450** |
| ***Streptococcus pasteurianus*** | 0 | 0 | 0 | 0 | 0 | 1 | **10** |
| ***Methylobacterium rhodesianum*** | 1 | 0 | 0 | 0 | 0 | 0 | **10** |
| ***Acinetobacter johnsonii*** | 0 | 0 | 0 | 0 | 0 | 1 | **29** |
| ***Rickettsia tamurae*** | 1 | 1 | 1 | 1 | 1 | 1 | **814** |
| ***Rickettsia endosymbiont*** | 0 | 1 | 0 | 1 | 1 | 0 | **43** |
| ***Spiroplasma sp.*** | 1 | 0 | 1 | 1 | 0 | 0 | **116034** |
| ***Rickettsia Rickettsia*** | 1 | 1 | 1 | 1 | 0 | 1 | **248630** |
| ***Neisseria zoodegmatis*** | 0 | 0 | 0 | 1 | 0 | 0 | **28** |
| ***Rickettsia bellii*** | 0 | 0 | 0 | 1 | 0 | 0 | **10** |
| ***Enhydrobacter aerosaccus*** | 0 | 0 | 1 | 0 | 0 | 0 | **10** |
| ***Rhodobacter capsulatus*** | 0 | 0 | 0 | 0 | 1 | 0 | **22** |
| ***Moraxella osloensis*** | 0 | 0 | 0 | 0 | 1 | 0 | **35** |
| ***Lactobacillus mucosae*** | 0 | 0 | 0 | 0 | 0 | 1 | **11** |
| ***Paenirhodobacter enshiensis*** | 0 | 0 | 0 | 0 | 1 | 0 | **13** |
| ***Corynebacterium sp.*** | 0 | 0 | 0 | 0 | 1 | 0 | **32** |
| ***Lactococcus lactis*** | 0 | 0 | 1 | 0 | 0 | 0 | **11** |
| ***Microbacterium ginsengisoli*** | 1 | 1 | 1 | 1 | 1 | 1 | **3467** |
| ***Wolbachia pipientis*** | 1 | 1 | 1 | 1 | 1 | 1 | **3E+06** |
| ***Aquabacterium parvum*** | 0 | 0 | 0 | 1 | 0 | 0 | **25** |
| ***Halomonas stevensii*** | 0 | 0 | 0 | 1 | 0 | 1 | **91** |
| ***Meiothermus ruber*** | 0 | 0 | 0 | 1 | 0 | 0 | **10** |
| ***Sphingomonas yabuuchiae*** | 0 | 1 | 0 | 0 | 0 | 0 | **48** |
| ***Moraxella porci*** | 0 | 0 | 0 | 1 | 0 | 0 | **31** |
| ***Elizabethkingia miricola*** | 0 | 0 | 0 | 0 | 1 | 0 | **16165** |
| ***Rhodococcus fascians*** | 0 | 0 | 0 | 0 | 1 | 0 | **10** |
| ***Cloacibacterium normanense*** | 0 | 0 | 0 | 1 | 0 | 0 | **11** |
| ***Cupriavidus necator*** | 1 | 1 | 1 | 1 | 1 | 1 | **2207** |
| ***Chryseobacterium chaponense*** | 0 | 0 | 0 | 0 | 1 | 0 | **95** |
| ***Brevundimonas vancanneytii*** | 1 | 0 | 0 | 1 | 1 | 1 | **115** |
| ***Mesorhizobium australicum*** | 0 | 0 | 0 | 1 | 0 | 0 | **15** |
| ***Pseudoxanthomonas mexicana*** | 0 | 0 | 0 | 1 | 0 | 0 | **11** |
| ***Staphylococcus felis*** | 0 | 0 | 0 | 0 | 1 | 0 | **46** |
| ***Bartonella sp.*** | 0 | 1 | 1 | 0 | 0 | 1 | **242** |
| ***Paenibacillus tundrae*** | 0 | 0 | 0 | 0 | 1 | 0 | **10** |
| **Total** | **33** | **28** | **33** | **47** | **40** | **37** |  |
